# Virus and Potential Host Microbes from Viral-Enriched Metagenomic Characterization in the High-Altitude Wetland, Salar de Huasco, Chile

**DOI:** 10.3390/microorganisms8071077

**Published:** 2020-07-20

**Authors:** Yoanna Eissler, Cristina Dorador, Brandon Kieft, Verónica Molina, Martha Hengst

**Affiliations:** 1 Instituto de Química y Bioquímica, Facultad de Ciencias, Universidad de Valparaíso, Gran Bretaña 1111, Playa Ancha, Valparaíso 2360102, Chile; 2 Laboratorio de Complejidad Microbiana y Ecología Funcional, Instituto de Antofagasta & Departamento de Biotecnología, Facultad de Ciencias del Mar y Recursos Biológicos, Universidad de Antofagasta, Avenida Universidad de Antofagasta s/n, Antofagasta 1240000, Chile; cristina.dorador@uantof.cl; 3 Centre for Biotechnology and Bioengineering, Universidad de Chile, Beaucheff 851 (Piso 7), Santiago 8320000, Chile; mhengst@ucn.cl; 4 Lab of Dr. Steven Hallam, University of British Columbia, Vancouver, BC V6T 1Z4, Canada; kieft1bp@gmail.com; 5 Departamento de Biología, Observatorio de Ecología Microbiana, Facultad de Ciencias Naturales y Exactas, Universidad de Playa Ancha, Avenida Leopoldo Carvallo 270, Playa Ancha, Valparaíso 2340000, Chile; veronica.molina@upla.cl; 6 HUB Ambiental UPLA, Universidad de Playa Ancha, Avenida Leopoldo Carvallo 200, Playa Ancha, Valparaíso 2340000, Chile; 7 Departamento de Ciencias Farmacéuticas, Facultad de Ciencias, Universidad Católica del Norte, Av Angamos 0610, Antofagasta 1270709, Chile

**Keywords:** virus, metagenome, bacteriophage, high-throughput sequencing, archaea, bacteria, high-altitude wetland, microbial diversity, freshwater

## Abstract

Salar de Huasco is a wetland in the Andes mountains, located 3800 m above sea level at the Chilean Altiplano. Here we present a study aimed at characterizing the viral fraction and the microbial communities through metagenomic analysis. Two ponds (H0 and H3) were examined in November 2015. Water samples were processed using tangential flow filtration to obtain metagenomes from which the DNA fraction of the sample was amplified and sequenced (HiSeq system, Illumina). The ponds were characterized by freshwater and the viral-like particles to picoplankton ratio was 12.1 and 2.3 for H0 and H3, respectively. A great number of unassigned viral sequences were found in H0 (55.8%) and H3 (32.8%), followed by the family Fuselloviridae 20.8% (H0) and other less relatively abundant groups such as Microviridae (H0, 11.7% and H3, 3.3%) and Inoviridae (H3, 2.7%). The dominant viral sequences in both metagenomes belong to the order Caudovirales, with Siphoviridae being the most important family, especially in H3 (32.7%). The most important bacteria phyla were Proteobacteria, Bacteroidetes and Firmicutes in both sites, followed by Cyanobacteria (H0). Genes encoding lysogenic and lytic enzymes (i.e., recombinases and integrases) were found in H0 and H3, indicating a potential for active viral replication at the time of sampling; this was supported by the presence of viral metabolic auxiliary genes at both sites (e.g., cysteine hydrolase). In total, our study indicates a great novelty of viral groups, differences in taxonomic diversity and replication pathways between sites, which contribute to a better understanding of how viruses balance the cycling of energy and matter in this extreme environment.

## 1. Introduction

Wetlands play an important role in many ecological contexts, including climate change, biodiversity, hydrology, and human health [1]; unfortunately, these ecosystems are constantly threatened by human activities [2,3]. As of 2009, 33% of global wetlands had been lost [4], hence their study is highly necessary. Salar de Huasco is a salt-flat or *Salar* in local language, located in the Chilean altiplano and classified as a high-altitude wetland under the Ramsar convention since 1996. Despite this denomination, it is subjected to human impact by tourism and mining activities, underlining the importance of performing baseline studies of the natural ecology of this wetland.

This ecosystem presents a large variety of environments, from transient ponds and streams to a main lake where flamingos and llamas cohabitate among other species [5]. This wetland also harbors a high microbial biodiversity [6,7] with a very active microbial community [8,9] that is adapted to extreme physicochemical conditions such as extensive daily temperature ranges (−15 to 20 °C) [10], intense irradiation (up to 1125 W/m^2^) [8], high evaporation rates (1260 mm/year) versus low precipitation rates (150 mm y^−1^) and a high variation in salinity conditions, from freshwater to hypersaline [11]. Bacteria communities are dominated by Bacteroidetes, Proteobacteria, Actinobacteria and Cyanobacteria among others [5,7,12,13]. Archaea are represented by Euryarchaeota, Crenarchaeota, Diapherotrites, Thaumarchaeota, and Woesearchaeota as the predominant phyla [7,13,14].

Concomitant with strong ecosystem variability and high prokaryote diversity, viral communities in the wetland are also expected to be widespread and diverse and play a pivotal role in ecosystem function. Viruses are the most abundant entities in the biosphere and have the potential to infect all domains of life, producing diseases, influencing biogeochemical cycles, facilitating gene transfer, and affecting the course of microbial evolution. One important mechanism of viral impact on the diversity and function of microbial populations is through the incorporation of auxiliary metabolic genes (AMGs) [15], which may modulate cellular activity by expressing enzymes that participate in C, N, P and S metabolic pathways [16,17,18,19].

Viral abundance is ecosystem-dependent and fluctuates from 10^3^ to over 10^9^ viruses mL^−1^ [20,21,22]. For instance, in freshwater and saline environments, the average of virus-like particles (VLP) mL^−1^ is 7.00 × 10^7^ and 4.99 × 10^8^, respectively, with a virus to picoplankton abundance ratio (VPR) ranging from 0.01 to 267.2 and 0.2 to 144.8 [21]. The only study conducted in the Altiplano area addressing viruses as modulators of the active microbial community is from Salar de Huasco [13]. This study covered from freshwater to saline habitats, where values for viral abundance (8.44 × 10^5^ to 4.78 × 10^8^ VLP mL^−1^) and VPR (2 to 351) represented the upper range of, or were even higher than, those found in other freshwater and saline environments.

Similar to oceanic, brackish or freshwater ecosystems, bacteriophage represent the largest group of viruses in wetlands [20,23]. Caudovirales is the most common order among dsDNA viruses found in wetlands [24,25], and studies conducted in high-altitude wetlands in China isolated and characterized several bacteriophages within the Caudovirales order and the families Myoviridae [26], Siphoviridae [27] and Podoviridae [28].

Nevertheless, overall, little is known about viral community composition in high-altitude wetlands despite the improvement in high-throughput environmental DNA sequencing techniques that have allowed for viral classification through bioinformatic methods [29,30]. Here we examine two viral-enriched metagenomes from freshwater ponds in a high-altitude wetland and classify DNA viruses, identify potential new bacteriophage and their putative hosts, describe genes encoding enzymes related to the lytic and lysogenic cycles, and classify AMGs related to metabolic pathways of the biogeochemical cycles in Salar de Huasco wetland. As a first report in viral genomic characterization in this habitat, our results emphasize the need to complement studies of bacteria and archaea communities with viral community characterization in order to understand microbe-driven elemental cycles in Salar de Huasco.

## 2. Materials and Methods

### 2.1. Sampling Sites

**Figure 1:**
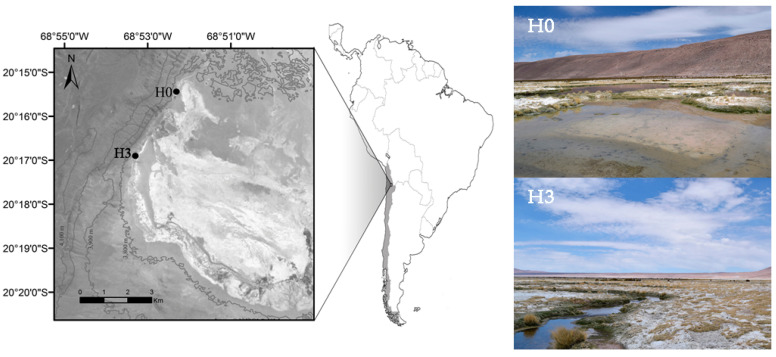
Map of Salar de Huasco, Chile, showing the location of both sampling sites (i.e., H0 pond and H3 stream) sampled during dry season in 10 November 2015.

Sampling took place in Salar de Huasco (3800 m a.s.l), Chile, during dry season in 10 November 2015, in two sites named H0 (20°15,904’ S; 68°52,401’ W) and H3 (20°16.983’ S; 68°53.342’ W) (Figure 1). H0 corresponded to a transient pond of approximately 30 cm deep and H3 to a stream connected to one of the spring waters that feed the Salar (Figure 1).

### 2.2. Sample Collection and Processing

Water temperature, conductivity, pH, and oxygen were measured in situ using a multiparameter Thermo Scientific Orion Star Multiparameter (model A329). Samples for phosphate analysis were frozen until processing using standard colorimetric methods with a nutrient automatic analyzer according to [31]. Samples (15 mL) for picoplankton and viral abundance were collected from each site and analyzed through the epifluorescence method [32]. Briefly, water was fixed with glutaraldehyde (1% final concentration) and stored frozen until analyses. Viral samples were first prefiltered with a Syringe Filter Unit, 0.22 µm, polyethersulfone (Millex_GP, Millipore^®^, Darmstadt, Germany) and then filtrated onto a 25 mm, 0.02 μm pore-size Anodisc membrane filters (Whatman^®^, Buckinghamshire, UK) at 130 mm Hg pressure. Filters were placed in the bottom of a Petri dish in darkness and stained for 15 min with 100 μL of SYBR^TM^ Gold Nucleic Acid Gel Stain (10,000×; Invitrogen^TM^, Carlsbad, CA, USA) diluted to a final concentration of 2× in filtered 0.02 μm molecular biology grade water. Filters were then blotted on a Kimwipe^®^ (Kimtech^science^, Little Rock, AR, USA) to remove excess fluid and then mounted between a glass slide and coverslip. Picoplankton abundance was estimated following the same protocol described above for viral abundance, but without prefiltering the sample and using a 0.2 μm pore-size Anodisc membrane filter (Whatman^®^, Buckinghamshire, UK). Ten to twenty fields were randomly selected and more than 100 VLP or picoplanktonic cells were counted per slide at 1000× using an Olympus BX60F-3 epifluorescence microscope, equipped with a HBO 50 W mercury lamp (excitation wavelength 460–490 nm, cutoff filter 515 nm).

To process water samples, tangential flow filtration (TFF) was used [33]. First, in order to remove large particles for TFF, 5–10 L of water underwent serial filtrations at 100 µm and 25 µm using nylon fiber sieves, then through a 0.22 µm Millipore^®^ Sterivex™ filter unit polyethersulfone membrane. To enrich the samples in virus-like particles (VLP), a 30 kDa spiral-wound TFF filter (Amicon S10Y30; Millipore^®^, Darmstadt, Germany) was used, followed by polyethylene glycol 8000 (Sigma-Aldrich, St. Louis, MI, USA) precipitation [34]. Then, the samples were treated with nucleases (2.5 U of DNase and 0.25 U of RNase) for 3 h at 37 °C to eliminate nucleic acids not encapsulated in viral capsids. Subsequently, a DNA extraction kit (ZR Viral DNA Kit™, Irvine, CA, USA) was used following the manufacturer’s instructions to extract viral DNA from capsids. The DNA obtained was quantified using a spectrophotometer (MaestroNano^®^, Maestrogen, Taiwan), and the genomic material was subject to non-specific amplification [35] using the GenomiPhi™ V2 DNA amplification Kit (Illustra™, GE Healthcare, Chicago, IL, USA) in order to obtain DNA concentrations required for sequencing. QIAamp^®^ DNA mini kit (QIAGEN^®^, Valencia, CA, USA) was used to further purify the DNA according to the manufacture’s protocol. Before sample sequencing, final DNA concentration was measured with MaestroNano^®^ spectrophotometer.

### 2.3. Sequencing and in Silico Analysis

The two DNA preparations were sequenced via Mr DNA-Texas service, using a HiSeq 2500 system (Illumina, USA). The paired-end reads from both H0 and H3 metagenomes were checked using the quality control of BaseSpace (https://basespace.illumina.com); these procedures yielded 11,614,382 reads totaling 2,558,493,699 base pairs (bps) with an average length of 220 bps (H0), and 10,096,566 reads totaling 2,230,955,051 bps with an average length of 221 bps (H3). Raw reads were submitted as forward and reverse fastq compressed files to the National Center for Biotechnology Information (NCBI) Sequence Read Archive (SRA) https://submit.ncbi.nlm.nih.gov/subs/sra/; the SRA BioProject number is PRJNA613071 and BioSample numbers are SAMN14395755 and SAMN14395756 for H0 and H3 raw reads, respectively.

Quality filtering was performed using Trimmomatic software Version 0.36 [36], with a quality cutoff score of 30 and a minimum length set at 50. The filtered sequences were assembled and contigs were obtained using SPAdes-3.11.1 [37] software in metaSPAdes mode with assembly parameters k: (21, 33, 55) and default settings. This Whole Genome Shotgun project has been deposited at DDBJ/ENA/GenBank under the accession JABXJQ000000000 and JABXJR000000000. The version described in this paper is version JABXJQ010000000 and JABXJR010000000 for H0 and H3 metagenomes, respectively.

Open reading frames (ORFs) and their location on all assembled contigs were predicted using the Prodigal software [38]. Quality-filtered paired-end reads were aligned to ORFs using bowtie2 [39]. Abundance values were calculated for each ORF based on read mapping using the transcripts per million (TPM) calculation [40]. Although this method was originally developed for cDNA transcripts, we applied the TPM calculation to DNA reads here in order to normalize ORF abundances by (1) predicted ORF length (in kilobase-pairs) and (2) library size (total DNA sequencing depth); this accounts for differences in these measures between H0 and H3. Thus, our TPM method resulted in an abundance metric of ORF-mapped metagenome reads per million reads in each sample. For ORF taxonomic and functional annotations, taxonomic accession numbers were assigned to ORFs using a diamond homology [41] search against the RefSeq protein database. The NCBI program entrez was used to assign taxonomy to accession numbers. Functional annotations for ORFs were made using the ghostKOALA tool [42] against the KEGG orthology database. Metagenome gene (ORF) TPM abundances were summed by their unique taxonomic string identifier (domain, phylum, class, order, family, genus, species) and used as the count table (in place of a standard OTU table) to estimate richness and alpha-diversity using the ‘vegan’ R package [43].

In order to compare the sequence identity of taxonomically unassigned contigs between the metagenomes (i.e., the similarity of the unannotated populations), first we chose contigs with no genes that could be assigned to a taxonomic group at the phylum level (i.e., either only domain was known or there was no classification at all). We used a sliding k-mer window of 31 bps along each contig to compare it (pairwise) to every other unassigned contig in both metagenomes. If two contigs had a Jaccard similarity index of >0.1 in k-mer space they were counted as “similar” (biologically, it was assumed they came from the same or a related population).

The assembled sequences were examined using Virsorter [29] to identify the function and phylogeny of viruses within the assembled metagenomes. For this, the platform CyVerse Discovery Environment (https://de.cyverse.org/de/) was used to run the application VirSorter 1.0.3 with the viromes database and decontamination mode. A list of contig sequences predicted as viral was obtained and organized by category from 1 to 6 (Tables S1 and S2). Here, we manually examined (Bioedit 7.2.6.1) and annotated the more confident viral predictions (categories 1—Pretty sure and 2—Quite sure) by using blastn suite online tool (https://blast.ncbi.nlm.nih.gov/). For H0 and H3, 299 and 217 nodes were manually examined, respectively. As a second and complementary approach, the program VIBRANT (v1.0.1) was used to predict viral contigs among all metagenome contigs [30]. In this unsupervised method, VIBRANT recovered 661 and 355 viral contigs from H0 and H3 assemblies, respectively (Tables S3 and S4), and flagged dozens of potential auxiliary metabolic genes (AMGs) on viral contigs.

## 3. Results and Discussion

Both H0 and H3 sites were characterized by low salinity, alkaline, and oxygenated water. Salinity was in the range of fresh water according to [44], which characterizes water sources as fresh when conductivity is <800 μS cm^−1^ (Table 1). These chemical characteristics were similar to those sampled during February 2012, November 2014 and March 2015 in Salar de Huasco [13,45]. VLP and picoplankton abundance were very similar in the H0 and H3 sites while VPR was slightly higher in H0 (Table 1). These values were lower than the ones observed in wet season, February 2012 [13] where the order of magnitude for VLP and picoplankton abundance was 10^7^–10^8^ VLP mL^−1^ and 10^5^–10^6^ cells mL^−1^, and the VPR was 67 and 2 for H0 and H3, respectively. Instead, during the sampling in November 2014 and March 2015, VLP (10^5^ VLP mL^−1^) and picoplankton (10^4^–10^5^ cells mL^−1^) abundance was lower than the ones report here, but VPR were similar to H0 (i.e., 9.4 and 13.2 for H0 spring and H3 spring, respectively) [45]. As reported in [13], where microbial mats were present, a higher abundance of picoplankton and VLP was observed, and this was also associated with higher microbial diversity and community complexity in ponds when compared with water sources or lagoon sites. During our study, microbial mats were scarce or not present at all, which might contribute to the lower abundance that was observed for viruses and picoplankton cells and the lower VPR.

Overall, VLP and picoplankton abundance values found in H0 and H3 during November 2015 were in the same range as those in other similar freshwater ecosystems [20,21,46]. Notably, however, a recent review [21] found an average of 7 × 10^7^ VLP mL^−1^ (*n* = 223) and 1.33 × 10^7^ cells mL^−1^ (*n* = 211) in freshwater ecosystems, which is higher in comparison to our results. Generally, high VPR values are related to high viral dynamics, low prokaryotic production, and/or high VLP abundance, and the ratio usually falls between 3 and 10 in aquatic ecosystems [20,46]. However, [21] reported an average of 17.2 (*n* = 299) for freshwater environments, suggesting that the lower VPR values observed in this study could indicate higher VLP loss or presumably higher microbial production. High viral losses have been associated with human activities, which can introduce chemicals and clays into the water that remove virus particles [47]. Salar de Huasco, despite it being a remote area, is still impacted by human activities; there is one small native family living close by that have livestock (llamas), and the area receives tourism traffic as well as mining exploration. Bacterial secondary production in this high-altitude wetland has been reported to be high [48] in comparison to other high-altitude lakes located in Europe [49,50,51]. Supporting these findings, Molina et al., (unpublished work) reported high secondary production in a pond located near H3 in November 2015, with values ranging from 2.56 to 4.24 μgC L^−1^ h^−1^ (data unpublished). Thus, the drivers of lower VPR ratios found in this study may be manifold, although the mechanisms were not directly tested.

### 3.1. Metagenome Composition

**Figure 2:**
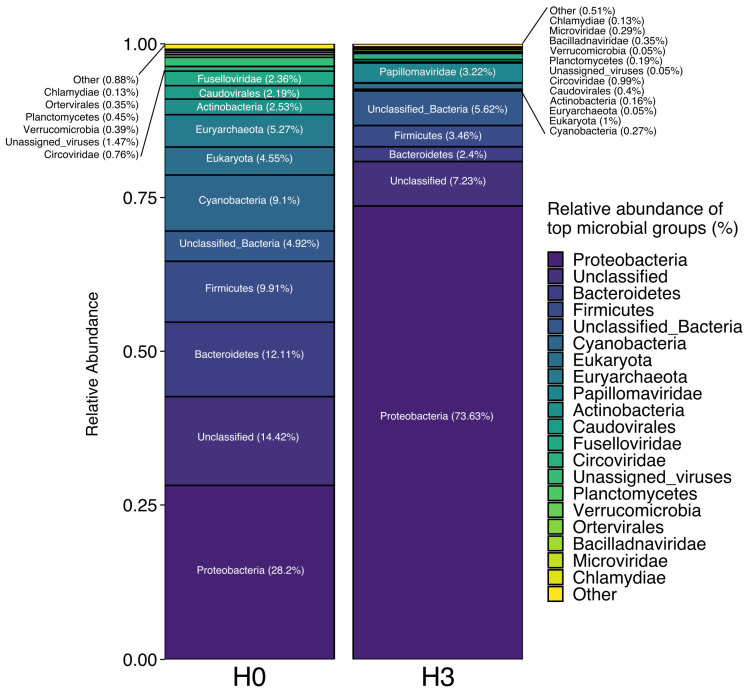
Total community taxonomic bar plot for H0 and H3 metagenomes.

Total taxonomic community composition (including both VLP and microbial populations) based on gene abundance (TPM) was markedly different between the two sites (Figure 2). H0 had a distinctly lower relative abundance of Proteobacteria than H3, but a much higher abundance of other Bacterial groups such as Bacteroidetes, Firmicutes, and Cyanobacteria. Total gene abundance in the H0 community was composed of over 5% Archaea and nearly 5% Eukaryotic populations, while in H3 the Archaea were represented at <0.05% and the Eukaryotes at <1%.

In general, the microbial community composition reported here is comparable with previous studies conducted in high-altitude wetlands [6,14]. In particular, a diversity survey carried out previously in three different ponds in Salar de Huasco [12] supports the dominance of Proteobacteria, Cyanobacteria and Bacteroidetes as core phyla present in all ponds, with Proteobacteria representing the majority of the prokaryotic community [12]. In our study we found a large difference in representation of Cyanobacteria between H0 (9.1%) and H3 (0.27%), in contrast to what was observed by [13], when Cyanobacteria were more highly represented in H3 than in H0. Interestingly, cyanophages were in low relative abundance in both sites but over twice as prevalent in H3, suggesting that virus–host dynamics might be influencing Cyanobacteria abundance over time in the ponds. Molina et al. [45] reported the three main abundant phyla of bacteria as Proteobacteria, Cyanobacteria and Bacteroidetes, and the abundant Archaeal phyla as Crenarchaeota, Parvarchaeota and Euryarchaeota based on 16S rDNA gene sequences. In contrast to our similar results for bacterial populations, these Archaeal phyla were relatively rare during November 2014 and March 2015 for H0 and H3 (<0.72%). Abundance may not be a strong indicator of importance, however, as when the active microbial community was studied using cDNA, the relative abundance of Archaea was much higher, reaching up to 30% and 70% in H0 and H3, with a greater contribution of Euryarchaeota and Thaumarchaeota phyla, respectively [13].

Supporting the patterns of taxonomic relative abundance presented in both sites, greater diversity and evenness was observed in H0 compared to H3 (Table 2). These observations are comparable to what was previously reported for pond sites in the same area during previous studies [13,45], where the highest alpha diversity indexes (i.e., Shannon index) were observed in ponds compared to waterways. Using a k-mer sequence similarity measure, we calculated that only 12.2% of contigs in the “unclassified” category from Figure 2 were shared between metagenomes, further highlighting the different in community composition between these two sites.

### 3.2. Viral Genome Composition

**Figure 3:**
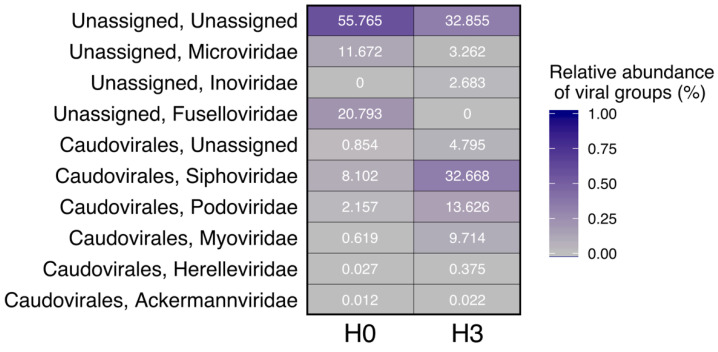
Viral taxonomy heatmap generated with VIBRANT.

Within the viral community identified by VIBRANT, each site appeared to host a distinct set of viral groups (Figure 3). Over half of viral contigs in the H0 community could not be assigned to a known group based on sequence homology, while only a third of viral sequences in the H3 community were unassigned. Since we did not recover enough DNA outright after sample processing, we used the multiple displacement amplification (MDA) method [35]. This random amplification technique requires attention while interpreting results, since it has some bias such as chimera formation [52], which could be interpreted as a novel sequence while in fact it is an artifact. Therefore, the percentage of unassigned sequences found in the sampling sites (Figure 3) might be overestimated. Unfortunately, there are no previous studies to compare viral diversity in high-altitude wetlands for the Altiplano area, but based on results of the prokaryotic community in our study and in others, there were a significant number of unclassified taxa, up to 56% in some cases [6,13,14,45], suggesting a high number of novel microbial groups and their viruses that are yet to be characterized.

Viral groups with strong differences in relative abundance between the sites included Inoviridae, Microviridae, Fuselloviridae, and several Caudovirales groups. Some putative hosts could be identified or inferred for these phage taxa, although the low resolution of viral phylogenetic assignment and the diverse nature of viral genomes provided only a coarse-scale putative host range identification in most cases (Table 3). When metagenomes are analyzed by sequence homology, the dependency of available sequences in databases must be taken into account; so far there is a very limited number of well-characterized viral species, most of them bacteriophage, and within this group there are also bias to dsDNA and Mycobacteriophage [53]. This could easily explain why there are mostly bacteriophage identified sequences through Virsorter and VIBRANT in our study, limiting comparison to only annotated viral sequences. A review that analyzed 12 freshwater metaviromes showed that viral sequences able to be identified were as low as 5.3% to 29.5% and the majority belong to phage that prey on bacteria [53].

Notably, one viral group targeting Archaea, the Fuselloviridae, had strikingly higher relative abundance in H0, corollary to the higher representation of its host domain Archaea at that site. This family of viruses infects mainly *Sulfolobus* sp. which belong to the phyla Crenarchaeota that have been previously characterized in H0 [46]. Furthermore, some other reports [54] show that Haloarchaea class members (phylum Euryarchaeota) may be hosts of crenarchaeal fuselloviruses, which were found to be very abundant in earlier studies in H0 [7,14].

In accordance with the higher relative abundance of Proteobacteria in H3, the abundance of several Caudovirales groups that most likely specialize in populations in the Proteobacteria phylum was much higher in the H3 metagenome. Table 3 also shows the identification of one viral sequence (7268 bps, 92% identity) in H3 belonging to Siphoviridae, which putative host is Actinobacteria. This phylum is only represented in H3 by a very small percentage (0.16%) and has also been reported before as a rare group in this wetland [45]. Microviridae, a family of bacteriophages with a single-stranded DNA genome, was relatively abundant among the assigned sequences, represented in H0 at 11.67% and in H3 at 3.26%. Single-stranded DNA viruses have been reported for freshwater ecosystems such as lakes [25] and the artic [24], but are not always well represented, like in the largest freshwater lake in Ireland where they constituted less than 1% of the virome [55]. In a thorough review of the literature we did not find metaviromes references from high-altitude freshwater wetlands for many viral sequences or groups that we identified, so it is not possible to provide an in-depth comparative analysis in this study.

### 3.3. Functional Analysis of Viral Sequences

**Figure 4:**
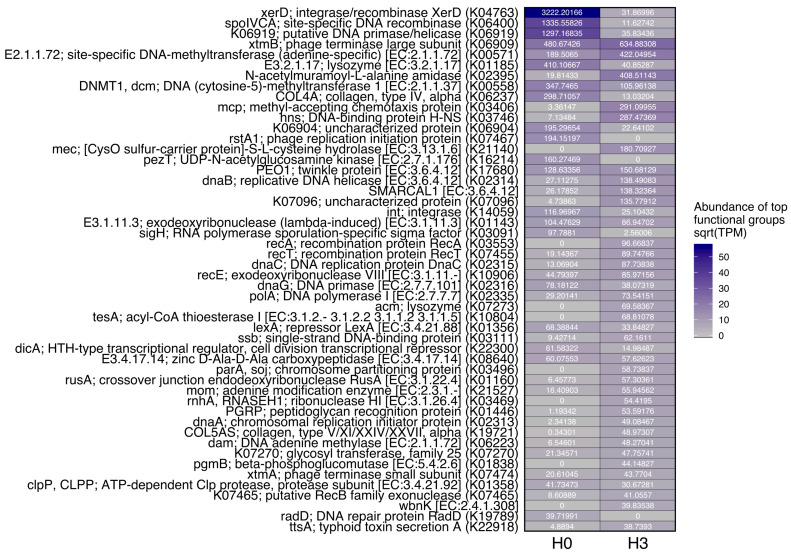
Viral genes heatmap generated with VIBRANT.

We expected that differences in phylogenetic representation of several ecologically important phage groups between the two sites would translate to a difference in the overall functional potential of the virome between samples. Based on the relative abundance of genes identified as viral in origin (by VIBRANT, measured as TPM), functional profiles of the H0 and H3 viral communities were highly distinct (Figure 4). Two recombinase genes (xerD and spolVCA) had the highest representation of all functions in H0. Interestingly, these genes were also recovered from H3 but at very low abundance compared to H0; this may indicate that a highly active phase of the lytic or lysogenic cycle was occurring during sampling from the H0 community since recombinase/integrase genes play an important role in integrating or excising viral DNA from a host genome [56]. Two functions strongly represented in the H3 viral community but not in H0 included enzymes responsible for cellular disruption (an amidase and a hydrolase), specifically, N-acetylmuramoyl-L-alanine amidase Rv3717, which is a cell-wall hydrolase that hydrolyzes the amide bond between N-acetylmuramic acid and L-alanine in cell-wall glycopeptides (UniProtKB - I6Y4D2). It may also be involved in the peptidoglycan degradation pathway [57,58], which is part of the cell wall degradation of several bacteria that occurs during a lytic cycle. In addition, H0 presented genes for the phage replication initiation protein and H3 did not (Figure 4), supporting the idea that one of the viral replication mechanisms was more predominant than the other; thus, lysogeny and lytic infections would be more dominant in H0 and H3, respectively.

**Figure 5:**
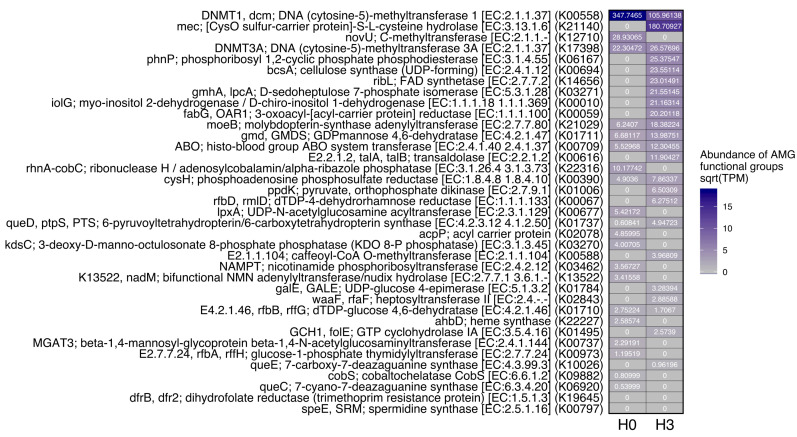
Viral auxiliary metabolic genes (AMGs) heatmap generated with VIBRANT.

Many AMGs encoded by the viral communities also had differences in representation between the two sites (Figure 5). A cysteine hydrolase AMG enzyme highly abundant in H3 but absent in H0 (cysO; [59]), along with the higher representation of the H3 autolysin enzymes mentioned above, supports the more lytic nature of phages in the H3 community during sampling.

Evidence shows that in freshwater environments, the relatively high availability of resources, such as carbon, nitrogen and phosphorus, seems to promote lytic cycles while the limitation of these elements produces lysogenic infection mechanisms [60,61]. In particular, when phosphorus is limited, lysis could be inhibited due to the paucity of phosphate to produce the backbone of the viral DNA structure [61,62]. In our study, phosphate concentration was 0.081 and 0.090 mg L^−1^ for H0 and H3, respectively which are similar to values reported earlier for this wetland [13,45] and are considered high for a freshwater environment [63]. This could indicate a more lytic nature in the infections occurring in the ponds studied. Although in both sites genes that encode for enzymes related to both types of replications mechanisms were found, in H3 the enzymes that participate in the wall cell degradation were much more abundant (Figure 4). In addition, in both sites, AMGs that encode for proteins that function in phosphate metabolism were found (e.g., phnP, gmhA, cysH, cobC and kdsC) (Figure 5). These types of genes have been also reported in other metagenomic studies in marine [64,65,66] and freshwater environments [56].

The viral replication mechanisms taking place in H0 and H3 could influence the way that microbial communities use carbon sources; for instance, during a lysogenic infection the host gene expression is modulated in such a way that its overall metabolism is reduced and the rate of inorganic and organic matter mineralization for growth is decreased [67,68], which affects the bulk carbon cycle and turnover of local nutrients. Further, Bonetti et al. [69] suggested that lysogenic and lytic cycles could decrease microbial growth efficiency and lead the pool of sequestered carbon in freshwater wetlands to be emitted as greenhouse gases. Future research should focus on resolving questions related to the abiotic and/or biotic modulators of viral infection (e.g., temperature, nutrients availability, seasonality and microbial diversity), the impact of viral phase on biogeochemical cycles, and the role of viral infection pathways and AMGs in carbon use, recycling of the organic matter, and emission of stored greenhouse gases in this wetland. Combined knowledge of these fundamental mechanisms will contribute to a better understanding of ecology in this fragile but polyextreme high-altitude wetland.

## 4. Conclusions

The metagenomes analyzed in this study contained a high level of unclassified prokaryote sequences, suggesting a high microbial novelty. In addition, one half and one third of viral contigs in the H0 and H3 sites, respectively, could not be assigned to a known group based on sequence homology, suggesting a high level of novel viral groups present in this high-altitude wetland. We suggest that there are many unknown phage–prokaryote relationships in this polyextreme environment. Among known sequences, the bacteria domain was composed mainly of Proteobacteria and Bacteroidetes, and, also to a lesser extent, phyla such as Firmicutes and Cyanobacteria. These groups showed differential abundance between the sampled sites.

Genes that participate in lysogenic and lytic pathways were present in the metagenomes analyzed for both sites and suggest that an active phase of the lytic or lysogenic cycle was occurring during the sampling time (November 2015, dry season), especially in H0 site. In comparison, H3 presented AMGs that encoded cysteine hydrolase, which has a catalytic activity that indicates a higher potential for lytic activity in this site at the time of sampling.

Viruses are biological entities that balance energy and matter cycling through ecosystems, and the knowledge obtained about these entities in our study provide a better understanding of ecosystems in high-altitude wetlands subjected to extreme conditions like Salar de Huasco.

## Figures and Tables

**Table 1:** Physicochemical parameters, viral, picoplankton abundance and virus to picoplankton ratio (VPR) determined for both sites.

Site	Conductivity (µS cm^−1^)	Water Temperature (°C)	pH	Dissolved Oxygen (mg L^−1^)	Phosphate (mg L^−1^)	Virus (VLP mL^−1^)	Picoplankton (cells mL^−1^)	VPR
H0	566.3	19.3	8.87	14.56	0.081	2.5 × 10^6^	2.1 × 10^5^	12.1
H3	580.7	17.6	8.68	8.32	0.090	1.3 × 10^6^	5.3 × 10^5^	2.3

**Table 2:** Metagenome richness and Alpha diversity indexes, including bacteria, archaea and viral diversity.

Site	Observed	Shannon	Chao1	Evenness
H0	2209	4.381	2346.1	0.00198
H3	1905	3.289	1918.5	0.00173

**Table 3:** Viral genome composition and structure diversity in according to Virsorter and VIBRANT.

Site	Node and Category	Size (bp) ^1^	Genome Structure	Features	Virus Group	Putative Host	VIBRANT ID; Viral Gene TPM
H0	278 and 1	8640 (3748)	linear	phage tail protein, phage tail tape measure protein, phage tail assembly protein, phage major tail tube protein	Caudovirales, Myoviridae, *Serratia* phage KSP20, 83.37% identity	Proteobacteria, Gammaproteo-bacteria (Enterobacteriaceae)	Peduovirinae; 28.39
H0	332, 581, 2647 and 1	8085 (3895), 6031 (2339), 2399 (1105)	linear	several hypothetical proteins	Myovirus (Uncultured Caudovirales phage clone similar to *Pseudomonas* phage), 72–81.45% identity	Proteobacteria, Gammaproteo-bacteria (*Pseudomonas* sp.)	Peduovirinae; 12.07 Peduovirinae; 32.26 Siphoviridae; 6.68
H0	5894 and 1	1425 (414, 268)	linear	terminase, portal protein, terminase large subunit	Siphoviridae (*Arthobacter* phage Gordon and *Streptomyces* phage), 69.74% and 67.52% identity	Actinobacteria	Too short; NA
H3	114 and 1	15881 (6652)	linear	tail fiber protein, tail length tape-measure protein, tail assembly protein K and I, tail attachment protein, tail protein, tail terminator protein, lipoprotein	Caudovirales; Siphoviridae, *Pseudomonas* phage phiAH14a, complete genome, 85% identity	Proteobacteria, Gammaproteo-bacteria (*Pseudomonas* sp.)	Siphoviridae; 1845.42
H3	1079 and 1	4431 (226)	circular	major capsid protein	O. Caudovirales, Fam Microviridae, 68.92% identity	Proteobacteria	Microviridae; 247.37
H3	1155 and 1	4188 (1772)	circular	terminase, head morphogenesis protein	Myovirus (Uncultured Caudovirales phage clone), 76% identity	Proteobacteria, Gammaproteo-bacteria (*Pseudomonas* sp.)	Siphoviridae; 22.545
H3	300 and 2	10081 (10275)	linear	phage major tail sheath protein, phage major tail tube protein, phage tail protein D and E	O. Caudovirales; Fam Myoviridae; Sub Fam Peduovirinae; Peduovirus; unclassified Peduovirus, (*Burkholderia* phage ST79) 92% identity	Proteobacteria, Betaproteobacteria	Peduovirinae; 49.34
H3	449 and 2	8201 (7268)	linear	exonuclease, DNA primase	O. Caudovirales; Siphoviridae; Pahexavirus; unclassified Pahexavirus, *Propionibacterium* phage pa28, complete genome, 92% identity	Actinobacteria	Siphoviridae; 107.52

^1^ number in parenthesis = size of the fragment that belongs to the viral identified sequence within the whole segment, NA = not available.

## Supplementary Materials

Supplementary materials can be found at https://www.mdpi.com/2076-2607/8/7/1077/s1, Table S1: VIRSorter_global-phage-signal_H0, Table S2: VIRSorter_global-phage-signal_H3, Table S3: H0_vibrant_results_full, Table S4: H3_vibrant_results_full.
